# Treatment Priorities in Patients With a History of Laryngeal Cancer: A Conjoint Analysis

**DOI:** 10.1002/ohn.70286

**Published:** 2026-06-17

**Authors:** Montana K. Upton, Sindhura Sridhar, Alexis Miller Dennison, Kavita Prasad, Thomas Issa, Rishi Kondapaneni, Elliot Abemayor, Abie H. Mendelsohn, Robert Sinard, Sarah Rohde, Patrick Tassone, Michael C. Topf

**Affiliations:** ^1^ Department of Otolaryngology–Head and Neck Surgery Vanderbilt University Medical Center Nashville Tennessee USA; ^2^ Department of Otolaryngology University of Alabama at Birmingham Birmingham Alabama USA; ^3^ Department of Otolaryngology–Head and Neck Surgery at Beth Israel Deaconess Medical Center Boston Massachusetts USA; ^4^ Department of Head and Neck Surgery UCLA Los Angeles California USA; ^5^ Department of Otolaryngology–Head and Neck Surgery University of Missouri Columbia Missouri USA

**Keywords:** conjoint analysis, counseling, larynx cancer, shared decision‐making

## Abstract

**Objective:**

To investigate patient priorities that may inform the choice of laryngeal cancer treatment.

**Study Design:**

Multi‐institutional, survey‐based, conjoint analysis study focusing on seven attributes: lifespan, treatment type, cancer cure, self‐image, mode of breathing, voicing, and swallowing. Patients with a history of treated laryngeal cancer (>6 months from treatment completion with no evidence of recurrent disease) were recruited.

**Setting:**

Tertiary care medical centers.

**Methods:**

Conjoint analysis yields utility scores, a quantitative measure of preference for an attribute. Higher utility scores indicate greater preference. Chi‐squared, univariate logistic regression, and univariate linear regression analyses were used to evaluate associations between patient demographic and medical features with relative attribute preference.

**Results:**

This study included 151 patients with previously treated laryngeal cancer. For the cohort, the mean importance scores (±standard deviation) were swallowing 25.7% (±8.4%), lifespan 21.5% (±9.3%), cancer cure 14.0% (±6.4%), mode of breathing 12.8% (±4.8%), voicing 9.2% (±3.5%), treatment type 9.1% (±5.0%), and self‐image 7.7% (±4.4%). Patients who required salvage surgery after upfront chemoradiotherapy placed more value on cancer cure compared to the other treatment groups (coefficient 2.76, 95% CI 0.33‐5.19).

**Conclusion:**

In patients with a history of treated laryngeal cancer, swallowing is the most important treatment priority, followed closely by lifespan. However, patients who underwent salvage surgery placed more value on cancer cure during decision‐making. These findings demonstrate that patient treatment preferences are diverse and may change throughout the cancer care journey, with some patients placing a higher value on quality of life than lifespan and cancer cure.

The publication of the 1991 landmark VA Laryngeal Cancer Study group trial established laryngeal preservation therapy as an acceptable alternative to surgical management in locally advanced disease, sparking a paradigm shift in the treatment of laryngeal cancer in the following decades.^1^ Tumor stage has a profound influence on prognosis, and T stage often substantially informs treatment decisions. For example, patients with advanced T4 tumors are likely to receive a survival benefit from upfront surgical management followed by appropriately selected adjuvant therapy.^2^, ^3^ In contrast, patients with T3 tumors may have equal oncologic outcomes but significantly different functional outcomes depending on the choice of upfront surgery or definitive chemoradiation therapy.^4^ Similarly nuanced decisions must often be made even with early‐stage cancer, where patients and providers must navigate ambiguity in expected oncologic and functional outcomes.

The decision between surgical management and chemoradiotherapy is complex due to the morbidities inherent to each treatment modality. Total laryngectomy results in loss of natural voice, creation of a permanent stoma, and alterations to physical appearance—all of which can lead to body image concerns, depressive symptoms, and psychological stress.^5^, ^6^, ^7^, ^8^ Alternatively, while chemoradiotherapy may allow for an anatomically intact organ, the resulting function is often suboptimal with a high risk of impaired speech, swallow, and breathing functions.^3^, ^5^ While there has been an emphasis on shared decision‐making within otolaryngology in recent years,^9^, ^10^ patients with head and neck cancer continue to experience clinically significant decisional conflict regarding their cancer treatment.^11^, ^12^, ^13^ In addition, patients with head and neck cancer have been shown to make decisions differently than patients with less severe illnesses, often deciding just “to do something” and place trust in their physician rather than choosing a specific treatment themselves.^14^, ^15^

Conjoint analysis is a well‐established market research strategy used to investigate consumer decision‐making. In recent years, it has been applied to a wide variety of medical decision‐making studies to improve patient clarity and decrease decisional conflict.^16^, ^17^, ^18^, ^19^ When using conjoint analysis, participants are presented with a series of choice tasks and asked to make trade‐offs between product options with varying feature, or attribute, levels. The data from these choice tasks is then used to assess the influence of each attribute and attribute level on the participant's decisions. We have previously utilized choice‐based conjoint analysis to investigate decision‐making for laryngeal cancer treatment in a healthy patient population, with findings demonstrating that the general public places the highest value in lifespan, voicing and swallow function, respectively.^20^ To our knowledge, there are no studies that use choice‐based conjoint analysis to investigate the priorities of patients who have completed treatment for laryngeal cancer. Thus, the aims of this study are twofold: (1) to understand the priorities of patients with a history of treated laryngeal cancer when choosing cancer treatment, and (2) to compare the priorities of patients with laryngeal cancer who underwent surgical management to those who received definitive chemoradiotherapy.

## Materials and Methods

This was a cross‐sectional, survey‐based, multi‐institutional study investigating patient decision‐making in laryngeal cancer treatment. Vanderbilt University Medical Center functioned as the coordinating institution, and patients were additionally recruited from the University of Missouri Health Center and the University of California at Los Angeles Health Center. Approval was obtained from the Vanderbilt University Institutional Review Boards, the UCLA Institutional Review Board, and the University of Missouri Institutional Review Board.

Patients were recruited from head and neck surgery clinics at each institution. Written consent was obtained. Patients were recruited for inclusion in the study if they had a history of treated laryngeal cancer and were >6 months out from treatment completion at the time of recruitment. Patients were excluded if there was concern for persistent or recurrent disease at the time of recruitment. Surveys were administered via portable tablet devices before, during, or after established return clinic visits. Research personnel were available during survey administration at all sites to assist patients and answer any questions. Sociodemographic information and pertinent medical and treatment history details were collected from the electronic medical records.

### Conjoint Analysis and Survey Construction

When using conjoint analysis, respondents are presented with a series of product options with experimentally varied features and asked to select the most preferable option. Investigators specify these features, also known as attributes, along with specific levels for each. Choice tasks are then created using these attributes and levels in an orthogonal design, which randomizes the presentation of the attributes and levels such that they are equally likely to be compared to each other. The resulting analysis, typically conducted using hierarchical Bayesian analysis, estimates attribute and level utility to model consumer decision‐making and product valuation.^21^, ^22^ The method of choice‐based conjoint survey description has been previously described in greater depth.^20^

**Figure 1:**
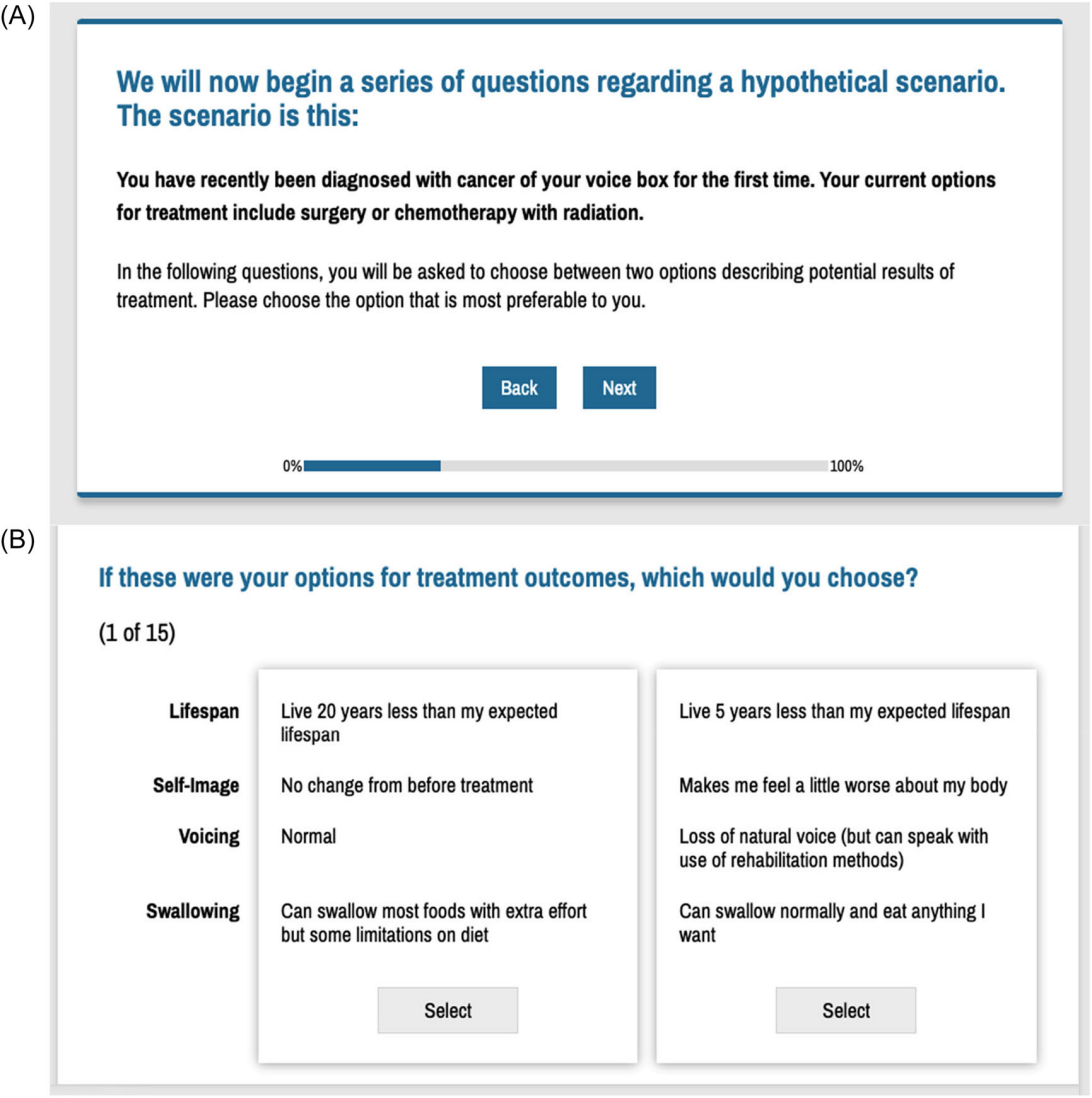
Hypothetical scenario presented to patients at the start of the survey (A) and an example choice task from a survey (B).

A conjoint analysis‐based survey was constructed using Sawtooth Software (Lighthouse Studio version 9.2.0). There were seven specified attributes of interest: lifespan, treatment type, cancer cure, self‐image, mode of breathing, voicing, and swallowing. Table 1 demonstrates the levels defined for each attribute. For continuity and continuation of prior work, the attributes from a prior study investigating laryngeal cancer treatment in a healthy noncancer population were used in this study.^20^ The attribute levels for Lifespan and Cancer Cure were consolidated in this study for clarity and to ensure the attribute levels had meaningful differences for patients. These attributes had previously been selected via review of existing literature and input from head and neck surgery faculty.^3^, ^6^, ^13^, ^23^ During the surveys, patients were asked to consider a hypothetical scenario in which they had received a new diagnosis of laryngeal cancer and then complete 15 choice tasks regarding potential treatment options for this new cancer. Figure 1 demonstrates the hypothetical scenario and example choice task from the survey. A sample survey was used to calculate the Flesch‐Kincaid grade level of 6.55, indicating the surveys on average required a reading level between sixth and seventh grades.

**Table 1:** Attributes and Levels for the Choice‐Based Conjoint Analysis

Attribute	Attribute levels
Lifespan	Live my expected lifespan. Live 5 y less than my expected lifespan. Live 10 y less than my expected lifespan. Live 20 y less than my expected lifespan.
Treatment type	Major surgery, with 1 wk in the hospital. Major surgery, with 1 wk in the hospital followed by radiation therapy for 5 wk. Chemotherapy with radiation therapy for 7 wk. Radiation therapy for 7 wk.
Cancer cure	90% chance of cure. 70% chance of cure. 50% chance of cure.
Self‐image	No change from before treatment. Makes me feel a little worse about my body. Makes me feel much worse about my body.
Mode of breathing	No limitations on physical activity. No limitations on physical activity, but will need a permanent breathing tube (tracheostomy). Some limitations on physical activity. Significant limitations on physical activity.
Voicing	Normal. Mildly hoarse voice (can talk on phone, but strained). Very hoarse voice (difficulty with phone conversations). Loss of natural voice (but can speak with use of rehabilitation methods).
Swallowing	Can swallow normally and eat anything I want. Can swallow most foods with extra effort but some limitations on diet. Will need a stomach feeding tube, but can still swallow a little food with effort. Cannot swallow anything, all nutrition goes through a stomach feeding tube.

### Statistical Analysis

Statistical analysis was conducted using Sawtooth Software (Lighthouse Studio Version 9.2.0) and R (R version 4.4.1, http://www.r-project.org). Descriptive statistics were used to summarize the clinical and demographic characteristics of the study cohort, stratified by treatment history. Pearson's chi‐squared tests were used to compare descriptive statistics between treatment groups.

Part‐worth utility scores (PWUSs), or quantitative measures of preference for each level of an attribute, were estimated using hierarchical Bayesian estimation.^24^ PWUSs were calculated for each individual respondent and averaged to generate a PWUS for each attribute level. Higher scores indicate increased relative preference for an attribute level.

Relative importance scores for each attribute were calculated by dividing the range of PWUSs for each attribute over the sum of the ranges of PWUSs for each attribute, resulting in a percentage score out of 100. Individual importance scores were averaged to generate a mean importance score for each attribute.

The effect of patient demographic and clinical characteristics on attribute utility score was calculated through univariate linear regression. For each patient characteristic, seven regression models were generated, one for each attribute. Patient characteristics assessed were age, gender, education (completed college or higher/no college), insurance (Medicaid/Medicare/Private), work status (working/not working), clinical T stage (T1, T2, T3, and T4a), salvage surgery (yes/no), history of a tracheostomy (yes/no), history of a feeding tube (yes/no), and overall stage (I, II, III, IVA, and IVB). Patients for which clinical or demographic information was missing were excluded from the regression analysis.

## Results

One‐hundred fifty‐one patients completed the larynx cancer decision‐making program. The study cohort included 114 males (75.5%), with a median age of 65 years (range 32‐90 years). The average time from treatment completion to survey was 2.9 years (range 12 days to 23 years). The cohort's tumor stage (T) was well balanced and included 45 patients (29.8%) with cT1 disease, 23 (15.2%) with cT2 disease, 42 (27.8%) with cT3 disease, and 37 (24.5%) with cT4a disease. There were four patients for which the T stage could not be determined from the chart review. Prior treatment history included 16 patients with radiotherapy only (10.6%), 35 with chemoradiotherapy (23.2%), 37 with chemoradiotherapy followed by surgery (24.5%), 26 with surgery only (17.2%), and 37 with surgery followed by chemoradiotherapy (24.5%). All patients who required surgery after chemoradiotherapy did so for recurrent disease, and no patients had a total laryngectomy for a dysfunctional larynx after chemoradiotherapy. In total, 84 patients (55.6%) had a history of a feeding tube during treatment, and 72 patients (47.7%) required a temporary or permanent tracheostomy. Table 2 demonstrates the complete demographic and clinical characteristics of the study cohort.

**Table 2:** Patient Demographic and Medical History Stratified by Treatment History

Characteristic	Chemoradiation (N = 35)	Chemoradiation + surgery (N = 37)	Radiation (N = 16)	Surgery (N = 26)	Surgery + chemoradiation (N = 37)	Overall (N = 151)
*Age, y*						
Mean (SD)	62.1 (9.99)	66.2 (9.96)	69.9 (11.6)	67.2 (11.0)	65.5 (8.08)	65.6 (10.1)
Median [min, max]	61.0 [32.0, 81.0]	67.0 [40.0, 85.0]	70.0 [44.0, 87.0]	66.0 [44.0, 90.0]	65.0 [49.0, 85.0]	65.0 [32.0, 90.0]
*Gender*						
Female	9 (25.7%)	14 (37.8%)	5 (31.3%)	3 (11.5%)	6 (16.2%)	37 (24.5%)
Male	26 (74.3%)	23 (62.2%)	11 (68.8%)	23 (88.5%)	31 (83.8%)	114 (75.5%)
*Work status*						
Not working	15 (42.9%)	21 (56.8%)	9 (56.3%)	11 (42.3%)	25 (67.6%)	81 (53.6%)
Working	20 (57.1%)	16 (43.2%)	7 (43.8%)	15 (57.7%)	12 (32.4%)	70 (46.4%)
*Education*						
Less than high school	3 (8.6%)	5 (13.5%)	2 (12.5%)	5 (19.2%)	5 (13.5%)	20 (13.2%)
High school	20 (57.1%)	21 (56.8%)	10 (62.5%)	15 (57.7%)	26 (70.3%)	92 (60.9%)
Associate's degree	4 (11.4%)	3 (8.1%)	2 (12.5%)	0 (0%)	1 (2.7%)	10 (6.6%)
Bachelor's degree	5 (14.3%)	5 (13.5%)	2 (12.5%)	3 (11.5%)	4 (10.8%)	19 (12.6%)
Professional degree	3 (8.6%)	3 (8.1%)	0 (0%)	3 (11.5%)	1 (2.7%)	10 (6.6%)
*Clinical T stage (cT)*						
T1	6 (17.1%)	7 (18.9%)	10 (62.5%)	12 (46.2%)	10 (27.0%)	45 (29.8%)
T2	7 (20.0%)	7 (18.9%)	3 (18.8%)	2 (7.7%)	4 (10.8%)	23 (15.2%)
T3	19 (54.3%)	12 (32.4%)	1 (6.3%)	3 (11.5%)	7 (18.9%)	42 (27.8%)
T4a	2 (5.7%)	11 (29.7%)	0 (0%)	9 (34.6%)	15 (40.5%)	37 (24.5%)
*Overall stage*						
I	2 (5.7%)	5 (13.5%)	8 (50.0%)	10 (38.5%)	10 (27.0%)	35 (23.2%)
II	3 (8.6%)	6 (16.2%)	3 (18.8%)	2 (7.7%)	1 (2.7%)	15 (9.9%)
III	13 (37.1%)	10 (27.0%)	1 (6.3%)	4 (15.4%)	5 (13.5%)	33 (21.9%)
IV (A and B)	12 (34.3%)	15 (40.5%)	0 (0%)	9 (34.6%)	20 (54.1%)	56 (37.1%)
*Feeding tube*						
No	20 (57.1%)	7 (18.9%)	14 (87.5%)	9 (34.6%)	11 (29.7%)	61 (40.4%)
Yes	15 (42.9%)	28 (75.7%)	1 (6.3%)	17 (65.4%)	23 (62.2%)	84 (55.6%)
*Tracheostomy*						
No	25 (71.4%)	10 (27.0%)	14 (87.5%)	13 (50.0%)	13 (35.1%)	75 (49.7%)
Yes	10 (28.6%)	27 (73.0%)	1 (6.3%)	13 (50.0%)	21 (56.8%)	72 (47.7%)

**Figure 2:**
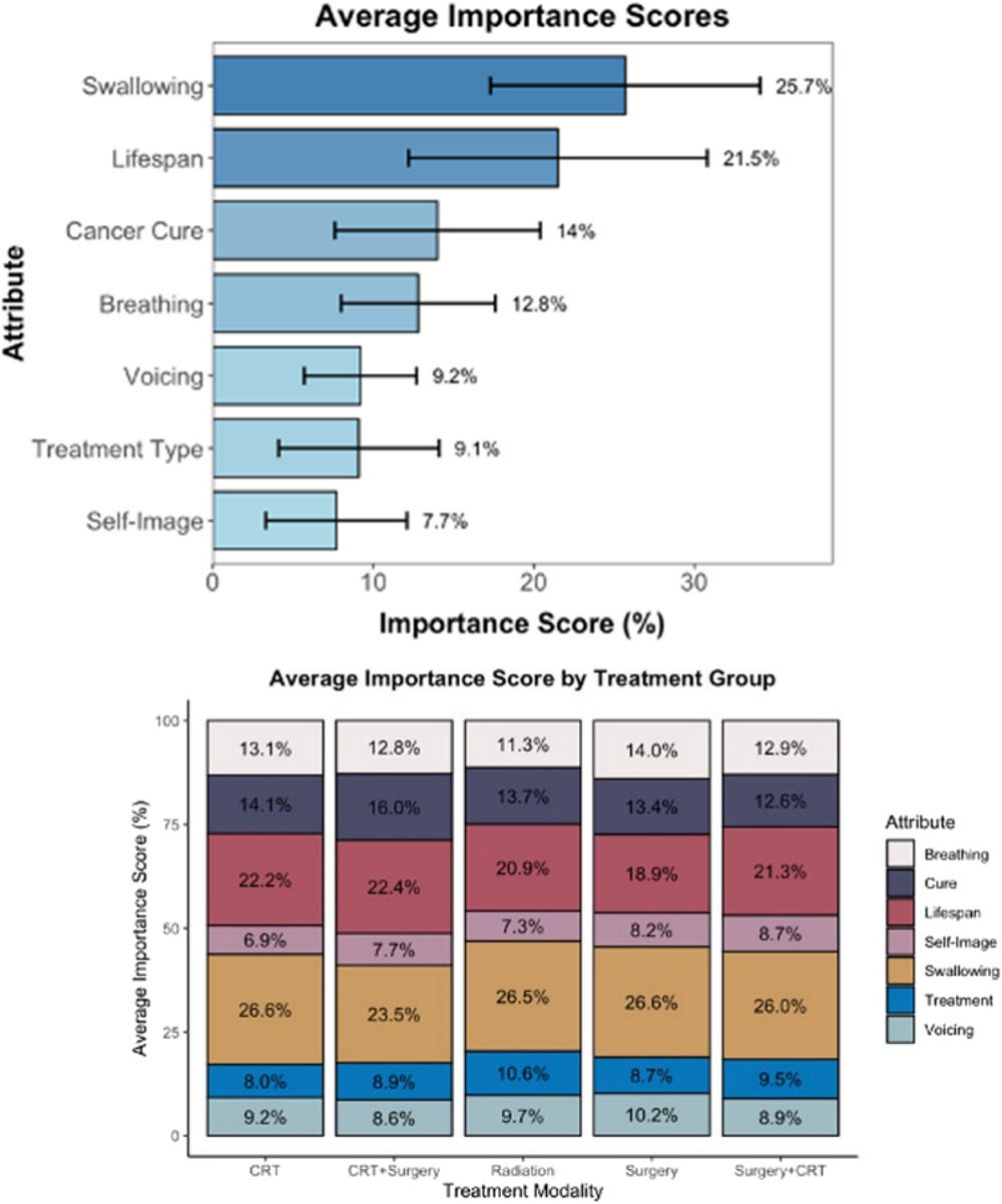
Importance scores for each attribute (A), and importance scores for each attribute stratified by treatment history (B). CRT, chemoradiation.

A summary of the attribute importance scores is displayed in Figure 2. For the entire cohort, the mean importance scores (±standard deviation) were swallowing 25.7% (±8.4%), lifespan 21.5% (±9.3%), cancer cure 14.0% (±6.4%), mode of breathing 12.8% (±4.8%), voicing 9.2% (±3.5%), treatment type 9.1% (±5.0%), and self‐image 7.7% (±4.4%).

When stratified by treatment group, swallowing and lifespan continued to have the highest relative importance scores compared to other attributes. A complete listing of the PWUSs for each attribute level, stratified by treatment history, is shown in Supplemental Figure S1, available online. A graphic demonstration of the PWUSs for each attribute level is demonstrated in Supplemental Figure S2, available online, with an increasing range in utility between the lowest and highest attribute level corresponding to increasing importance for that attribute.

Regression analysis showed that patients who required salvage surgery after chemoradiotherapy were more likely to value cancer cure during their decision‐making than the other treatment groups (coefficient 2.76, 95% CI 0.33‐5.19). Patients who were not employed were less likely to value treatment type (coefficient −1.72, 95% CI −3.30 to −0.14). Cancer cure was less important in older patients (coefficient −0.10, 95% CI −0.20 to 0.00). Patients with stage II disease at the time of treatment were less likely to value swallowing (coefficient −5.10, 95% CI −10.19 to −0.01). The complete regression analysis is displayed in Table 3.

**Table 3:** Linear Regression Analysis Investigating the Impact of Relevant Patient Factors and Medical History on Attribute Utility

Characteristic	Lifespan	Treatment type	Cancer cure	Self‐image	Mode of breathing	Voicing	Swallowing
*Age (coef, 95% CI)*	0.01 (−0.14, 0.16)	0.02 (−0.06, 0.10)	−0.10 (−0.20, 0.00)	−0.02 (−0.09, 0.05)	0.03 (−0.04, 0.11)	−0.01 (−0.06, 0.05)	0.07 (−0.07, 0.20)
*Gender (coef, 95% CI)*	−0.15 (−3.65, 3.35)	−0.40 (−2.26, 1.46)	1.18 (−1.21, 3.56)	0.96 (−0.68, 2.60)	−0.65 (−2.45, 1.16)	−0.52 (−1.84, 0.80)	−0.42 (−3.59, 2.74)
*Education (coef, 95% CI)*	−0.25 (−3.68, 3.19)	−0.01 (−1.84, 1.82)	0.38 (−1.97, 2.73)	−1.39 (−2.99, 0.21)	1.42 (−0.34, 3.18)	0.85 (−0.44, 2.14)	−1.00 (−4.11, 2.11)
*Insurance (coef, 95% CI)*							
Medicaid	1 [ref]	1 [ref]	1 [ref]	1 [ref]	1 [ref]	1 [ref]	1 [ref]
Medicare	−0.98 (−9.30, 7.34)	1.78 (−2.61, 6.17)	1.47 (−4.29, 7.23)	−1.70 (−5.66, 2.25)	0.78 (−3.63, 5.19)	0.13 (−3.03, 3.29)	−1.47 (−9.27, 6.34)
Private	−5.05 (−13.49, 3.39)	3.14 (−1.32, 7.59)	0.32 (−5.52, 6.16)	−1.74 (−5.76, 2.27)	1.23 (−3.24, 5.71)	1.23 (−1.98, 4.44)	0.88 (−7.04, 8.79)
*Work status (coef, 95% CI)*	2.11 (−0.89, 5.10)	−1.72 (−3.30, −0.14)	−0.44 (−2.51, 1.62)	−0.67 (−2.08, 0.74)	0.75 (−0.81, 2.30)	−0.36 (−1.50, 0.78)	0.34 (−2.39, 3.07)
*Clinical T stage (coef, 95% CI)*							
T1	1 [ref]	1 [ref]	1 [ref]	1 [ref]	1 [ref]	1 [ref]	1 [ref]
T2	3.90 (−0.77, 8.58)	−0.31 (−2.94, 2.23)	−0.62 (−3.86, 2.62)	−0.15 (−2.38, 2.07)	−1.09 (−3.53, 1.36)	0.32 (−1.49, 2.13)	−2.06 (−6.28, 2.17)
T3	0.40 (−3.51, 4.32)	−0.30 (−2.42, 1.83)	−1.87 (−4.59, 0.84)	1.40 (−0.46, 3.26)	−0.79 (−2.84, 1.26)	0.25 (−1.26, 1.77)	0.90 (−2.64, 4.43)
T4a	1.54 (−2.51, 5.59)	0.98 (−1.21, 3.18)	−1.08 (−3.89, 1.72)	0.21 (−1.72, 2.13)	−0.43 (−2.54, 1.69)	0.09 (−1.47, 1.66)	1.31 (−4.97, 2.35)
*Overall stage (coef, 95% CI)*							
I	1 [ref]	1 [ref]	1 [ref]	1 [ref]	1 [ref]	1 [ref]	1 [ref]
II	5.20 (−0.49, 10.88)	0.03 (−3.02, 3.08)	1.53 (−2.33, 5.39)	−0.71 (−3.42, 2.02)	−1.95 (−4.91, 1.01)	1.00 (−1.19, 3.19)	−5.10 (−10.19, −0.01)
III	0.88 (−3.59, 5.35)	−1.63 (−4.03, 0.77)	−1.00 (−4.03, 2.04)	1.09 (−1.05, 3.23)	0.11 (−2.21, 2.44)	0.54 (−1.18, 2.26)	0.01 (−3.99, 4.01)
IV (A and B)	1.75 (−2.22, 5.72)	−0.35 (−2.48, 1.78)	−0.82 (−3.51, 1.88)	0.32 (−1.58, 2.22)	−0.52 (−2.59, 1.55)	0.36 (−1.17, 1.89)	−0.75 (−4.30, 2.81)
*Salvage surgery (coef, 95% CI)*	1.46 (−2.13, 5.06)	−0.80 (−2.71, 1.11)	2.76 (0.33, 5.19)	−0.34 (−2.03, 1.36)	0.35 (−1.51, 2.21)	−0.62 (−1.97, 0.74)	−2.82 (−6.05, 0.41)
*History of tracheostomy (coef, 95% CI)*	0.44 (−2.62, 3.49)	0.94 (−0.68, 2.56)	0.30 (−1.78, 2.39)	−0.69 (−2.12, 0.75)	−0.98 (−2.56, 0.60)	0.26 (−0.91, 1.43)	−0.27 (−3.06, 2.53)
*History of feeding tube (coef, 95% CI)*	−1.04 (−4.17, 2.08)	1.58 (−0.06, 3.22)	−1.71 (−3.83, 0.40)	0.60 (−0.88, 2.07)	−0.61 (−2.24, 1.03)	0.02 (−1.16, 1.20)	1.17 (−1.67, 4.00)

## Discussion

This multi‐institutional study sought to better understand the priorities of patients with laryngeal cancer when deciding a course of cancer treatment by utilizing choice‐based conjoint analysis. Our cohort of patients with laryngeal cancer valued swallowing function above all other attributes, closely followed by lifespan. Voicing capabilities, treatment type, and self‐image all had relatively low value in comparison. We found no difference in treatment priorities between patients who underwent surgical management with or without adjuvant treatment and those who required chemoradiotherapy only. However, the patients who required salvage surgery after chemoradiotherapy were more likely to consider a cancer cure when making a decision compared to the other treatment groups.

An early study by McNeil et al sought to investigate the complex decision regarding loss of natural voice in laryngeal cancer by using a time‐trade‐off technique for utility analysis.^25^ They reported that a small percentage of their healthy volunteer population would trade some years of life to preserve their natural voice. Since this landmark paper, there have been a number of studies further exploring laryngeal cancer decision‐making. In a 2004 publication, List et al demonstrated that patients with newly diagnosed head and neck cancer would be more willing to tolerate aggressive treatments and undesirable side effects for prolonged survival compared to matched controls without a cancer diagnosis.^26^ In contrast, Laccourreye et al found that 63% of general otolaryngology patients would trade off a percentage chance of cure to preserve their larynx.^27^ However, the cohort of patients with cancer for List et al included all head and neck cancer subsites, with only 25% having a laryngeal primary, and Laccourreye et al surveyed a noncancer population. Hamilton et al showed that functional outcomes had a higher impact on value than treatment modality, and that head and neck cancer patients and providers had different values when considering laryngeal cancer treatment.^28^, ^29^ Similarly, a later study by Laccourreye et al found that 57.6% of otolaryngologists would consider trading some chance of cure to preserve their larynx.^30^

While such discrete choice experiments utilizing health states are helpful to investigate generalized preferences, they lack nuance to understand how the individual factors and outcomes that comprise health states may impact patient preferences. Conjoint analysis allows for a more nuanced investigation of priorities as it forces participants to make repeated trade‐offs and then determines which features are the most and least impactful when making those trade‐offs. The utility values established are, in turn, a proxy for the preferences or priorities of the population being tested. Such targeted investigations can be helpful to better understand head and neck cancer survivors, a vulnerable population with well‐established poor health‐related quality of life,^31^ as they allow patients to reflect on their own prior decisions and treatment experience to then establish these utility and importance scores.

We previously used conjoint analysis to examine laryngeal cancer treatment decision‐making in a healthy, noncancer population. This healthy population, with no personal or family history of head and neck cancer, predictably found lifespan to be most important during their decision‐making, followed by voicing and swallowing.^20^ While direct comparisons between separate conjoint analysis studies cannot be made given the nature of Bayesian analysis that is specific to each study population, it is interesting to note that the cancer patient population valued voicing capacity far less than the healthy population, ranking voicing as the fifth highest importance score compared to the healthy population's value as second‐most important. Both populations, however, placed a similarly low value on self‐image and treatment type. While these low importance scores do not mean that self‐image and treatment type hold no value to these patients, it does suggest that they hold relatively low importance when compared to the other attributes included in the studies. These findings are in contrast to work by Graboyes et al, who demonstrated high rates of body image disturbance in head and neck cancer survivors. However, these studies by Graboyes et al included patients with all head and neck cancer patients, with a high proportion of patients with oral cavity cancer.^32^, ^33^, ^34^ This may highlight that, in comparison, laryngeal cancer survivors are less concerned about self‐image.

Dysphagia is a well‐established complication of both surgical and non‐surgical laryngeal cancer treatment.^35^, ^36^ One large systemic review found rates of feeding tube dependence to be 32% to 100% during chemoradiotherapy, and up to 26% of patients required a long‐term feeding tube.^37^ This study also found high reported rates of post‐chemoradiotherapy aspiration (24%‐62%) and strictures (12%‐37%) leading to poor swallow function.^37^ Similarly, a systematic review of 44 articles examining postoperative laryngeal cancer treatment outcomes found the reported prevalence of dysphagia to be 35% to 89%, with symptoms including prolonged mealtime, need for multiple swallows, pain and coughing while eating, and regurgitation.^38^ Notably, Shuman et al found that patients with laryngeal cancer who prioritized eating and drinking prior to treatment and had lower scores on the MD Anderson Dysphagia Inventory survey post‐treatment had increased decisional regret about their treatment.^13^ In our own cohort of patients with laryngeal cancer, 55% required a feeding tube during their treatment. Thus, it is not surprising that the cancer patient population might retrospectively place such high importance on swallowing function over both lifespan and cancer cure. It is additionally possible that a lack of knowledge of swallow mechanics and how they relate to laryngeal cancer treatment may lead patients to be more surprised and negatively impacted by post‐treatment dysphagia. Most patients understand that treatment of a “voice box” cancer will lead to changes to their voice, but they may not know to expect such drastic changes to their swallowing. This places the burden of pre‐treatment patient education on all providers, including surgeons, medical and radiation oncologists, and speech language pathologists, to ensure appropriate counseling regarding expected post‐treatment outcomes.

While all treatment groups placed the highest value on swallowing and lifespan, patients who required salvage surgery were more likely to prioritize cancer cure during decision‐making than the other groups. Current data support 5‐year disease‐specific survival rates after salvage surgery for recurrent laryngeal cancer ranging from 48% to 57%, with low perioperative mortality, but increased risk of complications such as pharyngocutaneous fistula.^39^, ^40^, ^41^ However, there remains a paucity of data that focuses on understanding the priorities and functional outcomes of this select patient population. The results of this study suggest that the salvage surgery population is a distinct subgroup of patients with changing priorities as a result of their cancer treatment. These patients, who experience a more direct threat to life or lifestyle after failing initial chemoradiotherapy, experience a shift in their priorities with increasing importance of disease cure.

It is important to note some limitations when considering the findings of this study. Patients with a history of treated laryngeal cancer are influenced by their own treatment experience when completing the decision‐making program, unlike patients facing a new cancer diagnosis who have little to no knowledge of what they are facing. In addition, this study does not account for the treatment priorities of patients who have died from their disease or from other causes. Therefore, we may be misrepresenting the treatment preferences of patients with advanced laryngeal cancer that die from their disease. The patients included in this cohort came from three different tertiary academic centers across the country; while this produces diversity in the group, the findings still may not be applicable to all American patients or larynx cancer patients in other countries. There were a few patients who underwent transoral laser microsurgery as management of their disease included in this study, and we were not focused on examining preferences for this treatment type. The inclusion of expected lifespan as an attribute in this study varied from prior investigations, which focused on dying from cancer and not dying from cancer; we did not account for the differences in value that a prolonged lifespan may have based on the patient's age. Additionally, by the nature of conjoint analysis, participants are forced to relatively weigh the attributes against each other. However, in real life, some attributes could be considered almost evenly by patients. Finally, participation in this study necessitated that patients present for surveillance follow‐up. Thus, we did not capture the patient population who were lost to follow‐up and may have different priorities.

Overall, head and neck cancer providers should be aware that swallowing is important to patients who have undergone successful treatment for laryngeal cancer. However, as with most medical scenarios, patients remain complex and can have shifting values throughout their treatment course. This is most evident in the salvage laryngectomy group, with cancer cure gaining increased importance after failing upfront chemoradiotherapy. Moving forward, future studies would benefit from using a conjoint‐based platform to create a decision‐making tool that can be utilized in a prospective manner, allowing patients and providers to better understand individual priorities to then guide treatment decisions and set appropriate expectations for treatment outcomes.

## Conclusion

Patients with a history of treated laryngeal cancer found swallowing to be their highest priority when considering the choice of cancer treatment, closely followed by lifespan. Treatment modality and self‐image were the least valued treatment attributes. Although the majority of treatment subgroups had similar priorities, patients who required salvage surgery after upfront chemoradiotherapy placed a higher value in cancer cure. These findings suggest that patient priorities may differ from those of their providers, and emphasize the importance of open discussion between patient and provider to ensure appropriate expectations for post‐treatment outcomes.

## Authors' Note

This article was presented at the AHNS; July 2023; Montreal (oral presentation) and AHNS at COSM; May 2025; New Orleans (best poster in the “Hypopharynx & Larynx/Nasopharynx, Paranasal Sinus, & Skull Base Topics”).

## Author Contributions

**Montana K. Upton**, study design, analysis, drafted manuscript, presentation of the research; **Sindhura Sridhar**, data collection, analysis, drafted manuscript, presentation of the research; **Alexis Miller Dennison**, study design, data collection; **Kavita Prasad**, study design, data collection; **Thomas Issa**, data collection, manuscript editing; **Rishi Kondapaneni**, data collection, manuscript editing; **Elliot Abemayor**, study design, data interpretation; **Abie H. Mendelsohn**, study design, data interpretation; **Robert Sinard**, study design, data interpretation; **Sarah Rohde**, study design, data interpretation; **Patrick Tassone**, study design, manuscript editing/revision, data interpretation; **Michael C. Topf**, study design, manuscript editing/revision, data interpretation.

## Disclosures

### Competing interests

The authors declare no conflicts of interest.

### Funding source

This work was supported by a National Cancer Institute (NCI) K08 Career Development Award ‐ 1K08CA293255‐0.

## Supporting information


**Supplemental Figure S1**. Part‐worth utility scores for each attribute stratified by treatment history. **Supplemental Figure S2**. Part‐work utility scores for each attribute level, demonstrating the range in valuation the least and most desirable level for each attribute. Increased range between the lowest and highest attribute level correlates with increased importance of that attribute.
