# Supplementary figures and images for: Treatment Priorities in Patients With a History of Laryngeal Cancer: A Conjoint Analysis
Source: Otolaryngol Head Neck Surg. 2026 Jun 17;175(2):409–18. doi: 10.1002/ohn.70286 (PMC13417992; doi:10.1002/ohn.70286)

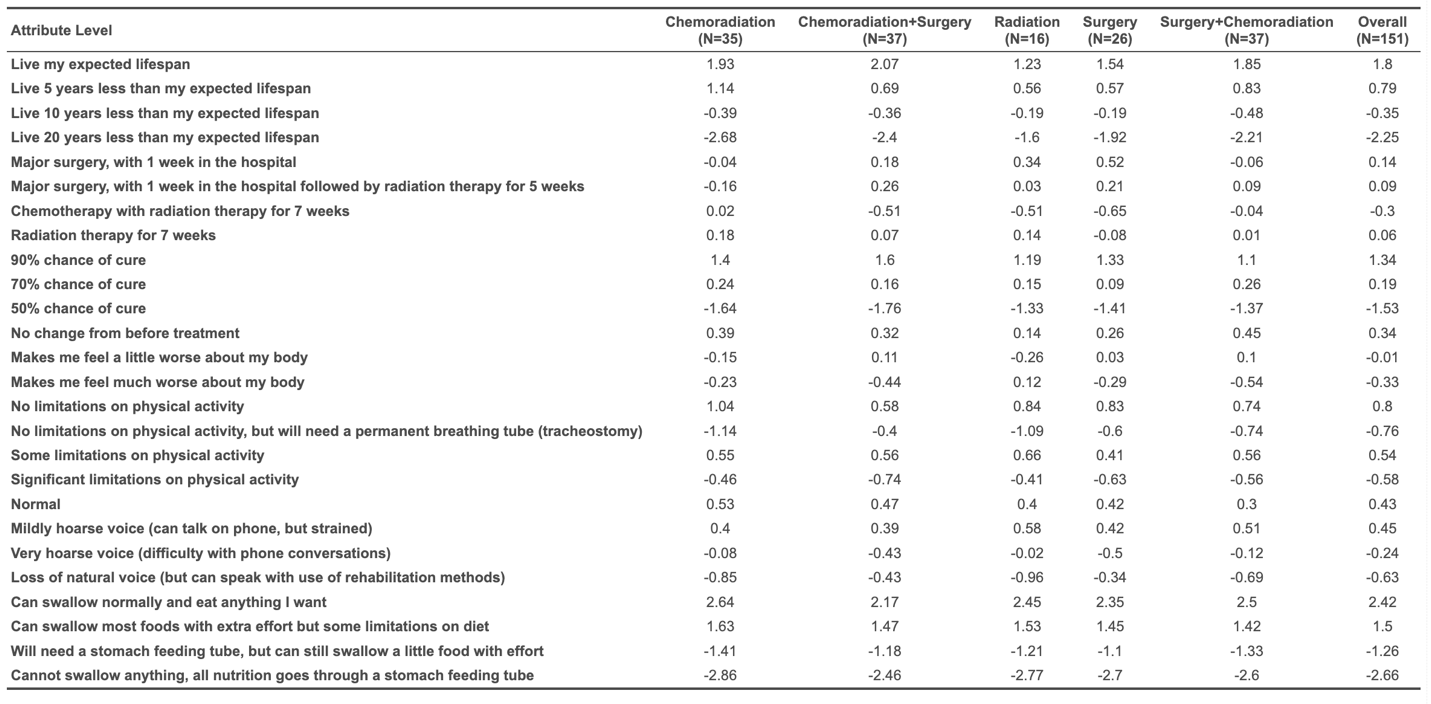


**Supplement 1**


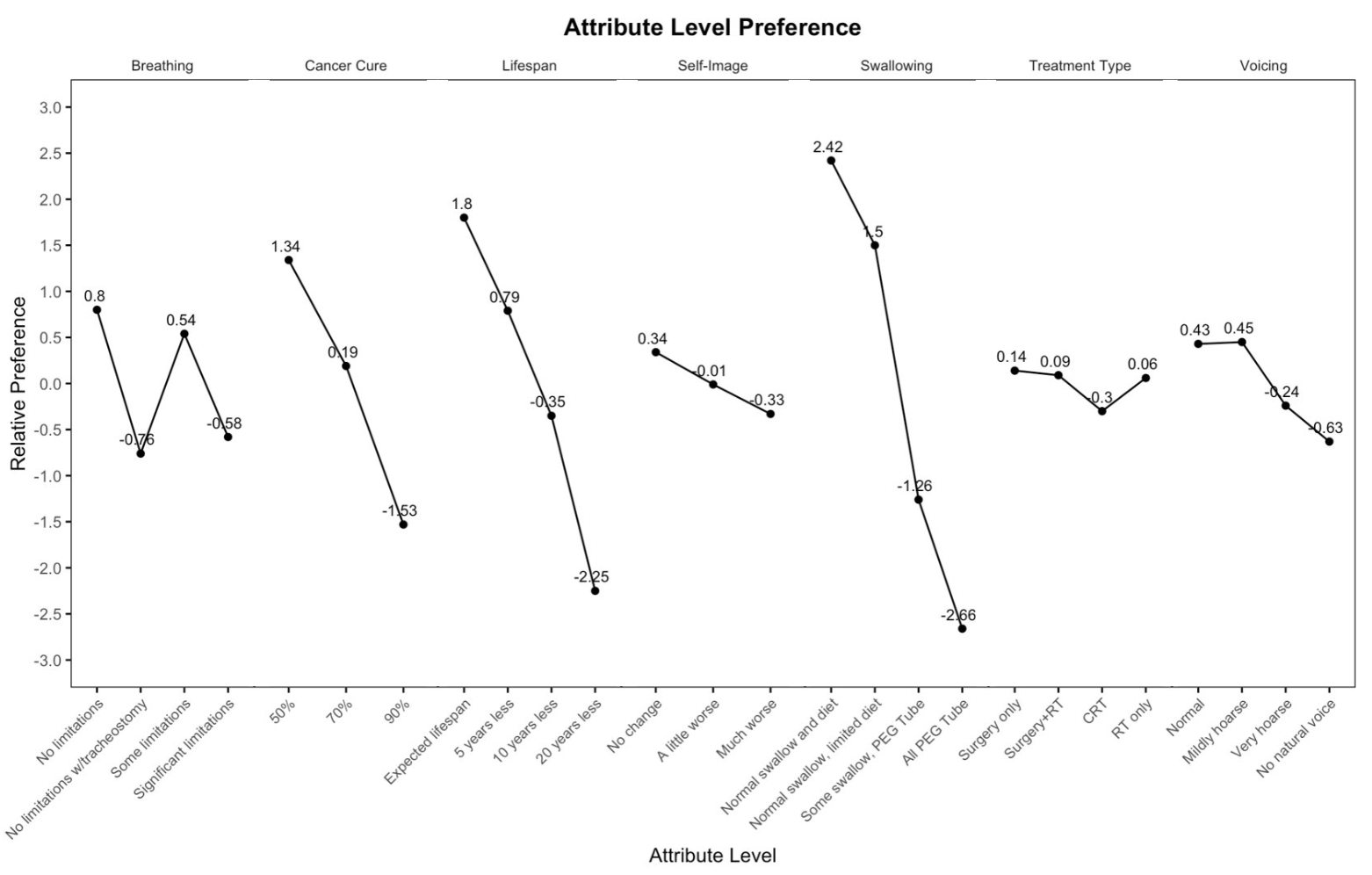


**Supplement 2**

Supplement: Supplementary file 1 — Supplemental Figure S1. Part‐worth utility scores for each attribute stratified by treatment history. Supplemental Figure S2. Part‐work utility scores for each attribute level, demonstrating the range in valuation the least and most desirable level for each attribute. Increased range between the lowest and highest attribute level correlates with increased importance of that attribute. [file OHN-175-409-s001.docx]
